# Analgesic and Functional Outcomes of Ultrasound-Guided Pericapsular Nerve Group (PENG), Fascia Iliaca, and Femoral Nerve Blocks in Hip Fracture Surgery: A Randomized Controlled Trial

**DOI:** 10.7759/cureus.103450

**Published:** 2026-02-12

**Authors:** Laxman Kumar Senapati, Rajendra Kumar Sahoo, Amrita Panda, Priyadarsini Samanta, Partha S Mohapatra, Subhadra Priyadarshini, Rajmohan Rao Tumulu

**Affiliations:** 1 Department of Anesthesiology, Kalinga Institute of Medical Sciences, Bhubaneswar, IND; 2 Department of Anesthesiology and Pain Medicine, Kalinga Institute of Medical Sciences, Bhubaneswar, IND; 3 Department of Physiology, Kalinga Institute of Medical Sciences, Bhubaneswar, IND; 4 Department of Biostatistics, Kalinga Institute of Medical Sciences, Bhubaneswar, IND

**Keywords:** analgesia, fascia iliaca compartment block, femoral nerve block, hip fractures, lateral femoral cutaneous nerve, pericapsular nerve group block, quadriceps muscle strength, ultrasound-guided regional anesthesia

## Abstract

Background and aim

Hip fractures are associated with significant pain and functional limitations in the perioperative period. Conventional regional anesthesia techniques, such as femoral nerve block (FNB) and fascia iliaca compartment block (FICB), provide analgesia but may cause quadriceps weakness. The pericapsular nerve group (PENG) block, a newer motor-sparing technique, may offer improved outcomes. This study aimed to compare the analgesic efficacy and functional outcomes of PENG + lateral femoral cutaneous nerve (LFCN) block with FICB and FNB + LFCN in patients undergoing hip fracture surgery.

Methods

In this randomized, assessor-blinded clinical trial, 180 patients with proximal femur fractures were assigned to PENG + LFCN, supra-inguinal FICB, or FNB + LFCN (n = 60 each). Blocks were performed under ultrasound guidance using standardized doses of ropivacaine and dexamethasone. The primary outcome was the median reduction in Visual Analog Scale (VAS) score during movement at 30 minutes post-block. Secondary outcomes included ease of spinal positioning, postoperative VAS scores, time to first rescue analgesia, 24-hour opioid consumption, quadriceps strength, patient satisfaction, and complications. Data were analyzed using the Kruskal-Wallis test, with post hoc pairwise comparisons performed using the Dwass-Steel-Critchlow-Fligner procedure.

Results

The median reduction in post-block VAS at 30 minutes was greatest in the PENG group (7.0 points), followed by the FICB group (5.0 points) and the FNB group (3.0 points) (p < 0.001). Post hoc analysis showed that PENG provided superior analgesia compared with both FICB and FNB (p < 0.0001), and FICB was also superior to FNB (p < 0.0001). The PENG group also had a longer median time to first rescue analgesia (24 hours; IQR: 12-24) and lower 24-hour median opioid consumption (5 mg morphine equivalent; IQR: 0-5) compared with the FICB and FNB groups (p < 0.001 for both). Patients in the PENG group demonstrated minimal quadriceps weakness and higher satisfaction (p < 0.001 for both). No major complications occurred.

Conclusions

The PENG + LFCN block provided superior analgesia, facilitated spinal anesthesia positioning, reduced opioid requirements, and better preserved quadriceps strength compared with FICB and FNB + LFCN. These findings support the PENG block as an effective motor-sparing alternative for perioperative analgesia in hip fracture surgery.

## Introduction

Hip fractures and the associated pain are significant health problems, particularly in the elderly population, due to multiple comorbidities and related complications [1]. Inadequately controlled pain in this population is associated with delirium, impaired pulmonary function, delayed mobilization, thromboembolic events, and prolonged hospitalization, all of which contribute to increased morbidity and poorer functional outcomes [2]. Hip surgery typically causes moderate-to-severe pain, which, if poorly managed, is linked to a higher risk of anxiety, cognitive impairment, delayed ambulation, thromboembolic events, and extended hospital stays [2]. Providing optimal sitting positioning for spinal anesthesia (SA) is also challenging because of fracture-related pain; inadequate positioning may delay anesthetic initiation, prolong procedural time, and increase patient discomfort and distress [3].

Regional anesthesia (RA) techniques are often favored due to their ability to provide superior analgesia while minimizing the systemic adverse effects of opioids, which are commonly used for postoperative pain management [4]. Femoral nerve block (FNB) and fascia iliaca compartment block (FICB) have been extensively used for hip fractures; however, postoperative quadriceps weakness remains a notable drawback [5,6]. The pericapsular nerve group (PENG) block is a recently described approach that exhibits motor-sparing effects and is gaining popularity [7,8]. Incorporating a lateral femoral cutaneous nerve (LFCN) block into the PENG block may improve analgesic outcomes by providing additional coverage of the lateral thigh skin incision area [9].

Evidence suggests that FNB and FICB are effective in alleviating pain in patients with hip fractures; however, direct comparisons between the newer PENG block and the established FNB and FICB are limited [10]. The primary objective of this study was to compare early analgesic efficacy among the three block techniques at 30 minutes post-block, measured using the Visual Analog Scale (VAS) during passive limb movement prior to SA. This time point was chosen because it directly influences positioning comfort and anesthetic workflow. Secondary objectives included assessment of postoperative pain at rest and during movement, ease of positioning for SA, time to first analgesic request, postoperative opioid consumption, quadriceps muscle strength, patient satisfaction, and adverse events. These outcomes were prespecified to evaluate both analgesic effectiveness and functional recovery. We hypothesized that the PENG + LFCN block would provide superior analgesia with motor-sparing effects compared to FICB and FNB + LFCN.

## Materials and methods

Study design, trial registration, and ethical consideration

This randomized controlled trial was conducted between February 25, 2022, and June 1, 2023, following approval from the institutional ethics committee (approval number KIIT/KIMS/IEC/767/2021, dated November 15, 2021) and registration with the Clinical Trials Registry- India (CTRI/2022/02/040557, dated February 23, 2022, www.ctri.nic.in). All participants provided written informed consent to participate.

Eligibility criteria

Patients aged 40 years and older, with American Society of Anesthesiologists (ASA) physical status I to III, and with proximal femur fractures (neck of femur or intertrochanteric) undergoing surgery under SA were included. Exclusion criteria were communication difficulties, old fractures (>7 days), polytrauma, surgery duration exceeding 2.5 hours, surgery not performed within 24 hours after block, conversion to general anesthesia after SA, or contraindication to RA or SA. Eligible patients were screened at hospital admission, and written informed consent was obtained prior to randomization and block administration.

Sample size estimation

Sample size estimation was based on the primary outcome: reduction in VAS score during movement at 30 minutes post-block. Effect size estimates were derived from prior randomized trials comparing two ultrasound-guided blocks, which reported between-group differences of approximately 2-3 VAS points [11,12]. A minimal clinically important difference of 2 VAS points was considered clinically meaningful [13]. For a three-group comparison, a one-way ANOVA framework was assumed, corresponding to an estimated Cohen’s f of 0.35. To account for the three planned pairwise comparisons, a Bonferroni-adjusted alpha level of 0.0167 (0.05/3) was applied. This adjustment was used solely for conservative sample size planning and did not influence the analytical post hoc method, for which the Dwass-Steel-Critchlow-Fligner (DSCF) procedure was employed to control the family-wise error rate. Under these assumptions, a minimum of 44-50 patients per group was required to achieve 80% power. To preserve power after multiple-comparison adjustment and account for uncertainty in effect-size estimation, 60 patients per group were enrolled (total n = 180). This sample size exceeded the requirements for both ANOVA and the Kruskal-Wallis test, confirming adequate statistical power.

Randomization, allocation concealment, and blinding

Randomization and allocation concealment were performed by an independent researcher (PSM) who had no role in patient care, block performance, or outcome assessment. Group allocation was generated using a computer-based randomization sequence and implemented using sequentially numbered, sealed, opaque envelopes. Ultrasound-guided nerve blocks were performed by a designated anesthesiologist (LKS), while postoperative outcome assessments were conducted by a separate blinded assessor (PS).

Postoperative outcomes, including pain scores, quadriceps muscle strength, opioid consumption, and patient satisfaction, were assessed by an anesthesiologist blinded to group allocation. Patients were not informed of the specific block technique administered. Due to the nature of the interventions, the anesthesiologist performing the nerve blocks could not be blinded; however, all postoperative assessments were conducted independently by a blinded anesthesiologist. Outcome assessors had no access to procedural details or ultrasound images. Standardized assessment protocols and postoperative analgesic regimens were applied across all groups to minimize performance and assessment bias. All blocks were performed by anesthesiologists with established experience in ultrasound-guided RA, each routinely performing these techniques in clinical practice. Standardized procedural protocols were applied to maintain technical consistency across study groups.

Intervention

**Figure 1:**
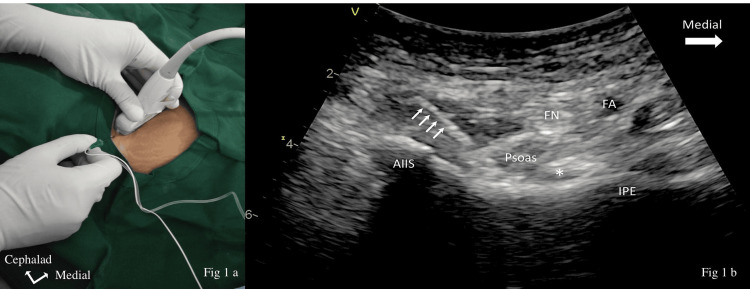
(a) Position of the hip, probe orientation, and needle insertion for the PENG block. (b) Sonoanatomy of the PENG block with the needle (white arrow) inserted from lateral to medial. The tip is located below the psoas tendon (asterisk) AIIS, anterior inferior iliac spine; FA, femoral artery; FN, femoral nerve; IPE, iliopubic eminence; PENG, pericapsular nerve group

**Figure 2:**
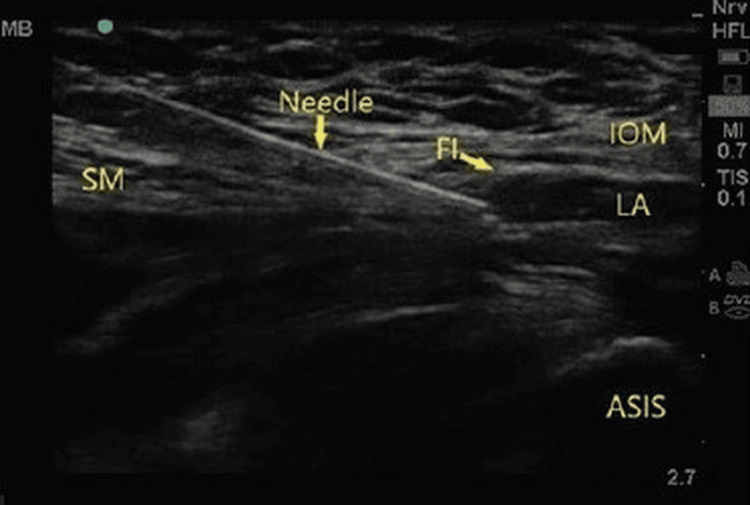
Sonoanatomy of the S-FICB showing the spread of LA below the FI ASIS, anterior superior iliac spine; FI, fascia iliaca; IOM, internal oblique muscle; LA, local anesthetic; S-FICB, supra-inguinal fascia iliaca compartment block; SM, sartorius muscle

**Figure 3:**
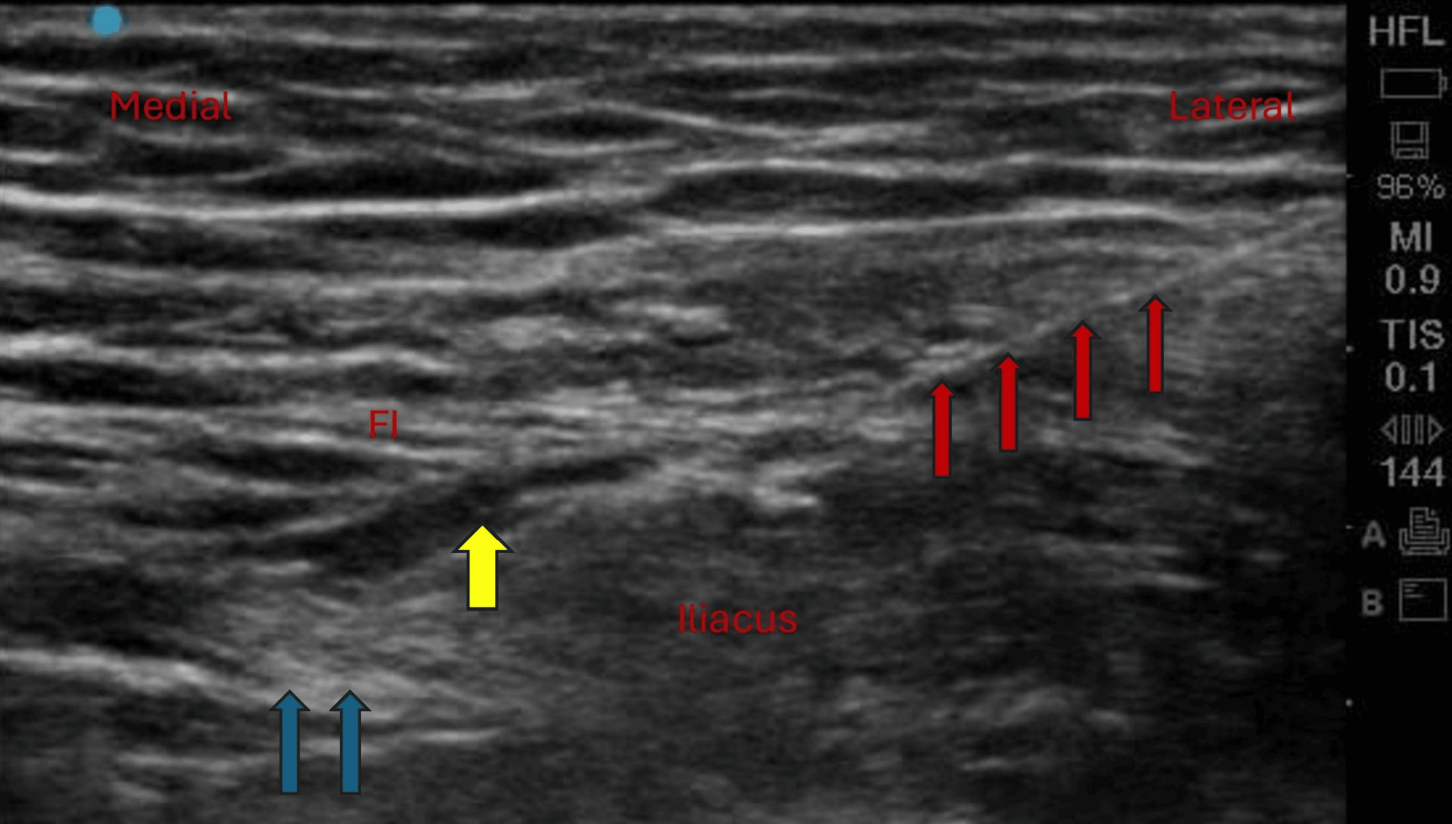
Sonoanatomy of the FNB showing the spread of LA (yellow arrow) around the FN (blue arrow), with the needle directed from lateral to medial (red arrow) FI, fascia iliaca; FN, femoral nerve; FNB, femoral nerve block; LA, local anesthetic

**Figure 4:**
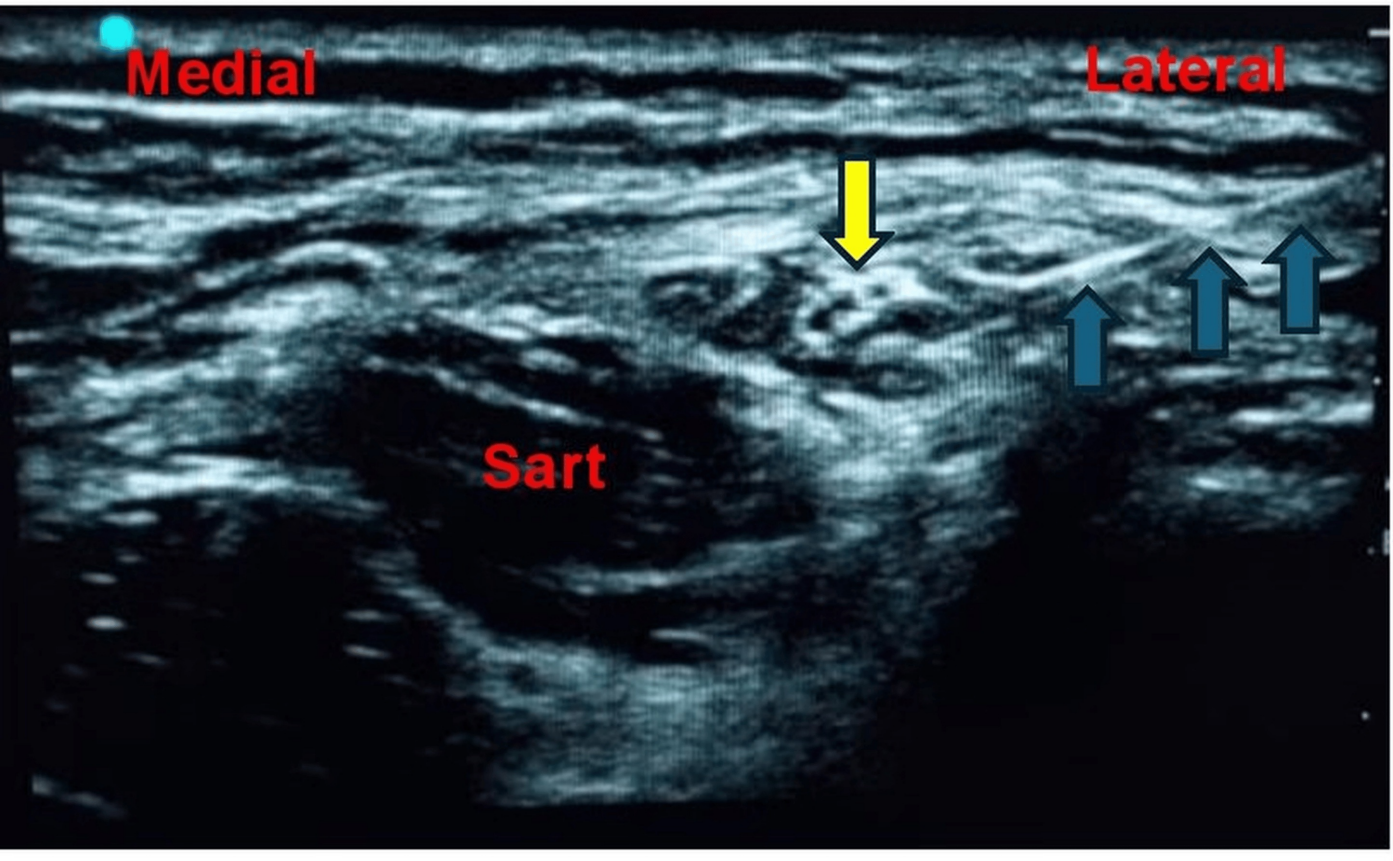
Sonoanatomy of the LFCN block showing the nerve (yellow arrow) at the lateral margin of the sartorius muscle, with block needle insertion (blue arrow) from lateral to medial and spread of LA around the LFCN LA, local anesthetic; LFCN, lateral femoral cutaneous nerve; SART, sartorius muscle

Patients in Group I (PENG + LFCN) received an ultrasound-guided PENG block with 25 mL of 0.25% ropivacaine combined with dexamethasone 4 mg (1 mL), followed by an LFCN block using 5 mL of 0.25% ropivacaine without dexamethasone. Patients in Group II (S-FICB) received an ultrasound-guided supra-inguinal FICB with 40 mL of 0.25% ropivacaine combined with dexamethasone 4 mg (1 mL). Patients in Group III (FNB + LFCN) received an ultrasound-guided FNB with 25 mL of 0.25% ropivacaine combined with dexamethasone 4 mg (1 mL), followed by an LFCN block using 5 mL of 0.25% ropivacaine without dexamethasone. In all groups, dexamethasone was added exclusively to the primary block injectate. All blocks were performed under ultrasound guidance (SonoSite Edge II, Bothell, WA, USA) using a linear high-frequency (6-13 MHz) probe for FICB, FNB, and LFCN blocks, and a curvilinear probe (2-5 MHz) for the PENG block, following established techniques (Figure 1, Figure 2, Figure 3, Figure 4).

Surgical management

Surgical procedures were performed according to standard institutional protocols based on fracture type. Patients with neck of femur fractures underwent hemiarthroplasty or total hip arthroplasty, whereas those with intertrochanteric fractures were managed with either dynamic hip screw fixation or proximal femoral nailing. All surgeries were conducted by the same orthopedic surgical unit using standardized perioperative and postoperative care pathways.

Anesthetic management

Preprocedural pain was assessed at rest (in the patient’s most comfortable position) and during passive movement (15° elevation) of the affected limb. Pain was evaluated using a VAS, consisting of a 10-cm (100-mm) line with “no pain” at 0 cm and “worst imaginable pain” at 10 cm. Thirty minutes after the blocks, VAS scores were recorded at rest and during passive limb elevation. The ease of spinal positioning (EOSP) was assessed using a scale from 0 to 3, where 0 indicates inability to position, 1 signifies abnormal posturing due to pain requiring support, 2 reflects mild discomfort without the need for support, and 3 represents optimal positioning allowing the patient to sit without pain [3]. SA was administered in the sitting position at the L3-L4 or L4-L5 interspace using 2-3 mL (10-15 mg) of commercially available 0.5% hyperbaric bupivacaine hydrochloride in dextrose injection USP (ANAWIN Heavy^®^, Neon Laboratories Limited, Mumbai, India), providing a solution denser than cerebrospinal fluid for predictable intrathecal spread. The dose was selected within this standardized range based on routine clinical judgment, considering patient characteristics such as age and body habitus. All patients received paracetamol (1 g IV) prior to surgical closure and three times daily thereafter as part of multimodal analgesia. Rescue analgesia with tramadol 50 mg IV was administered if the VAS score was ≥ 4, with an additional 50 mg given after 30 minutes if pain persisted. The total tramadol dose was limited to a maximum of 200 mg within 24 hours. Antiemetic therapy was standardized across groups, with ondansetron administered as clinically indicated to manage tramadol-related nausea or vomiting. Complications, including nausea, vomiting, local anesthetic systemic toxicity, vascular puncture, and nerve injury, were documented. Time to first rescue analgesia and total opioid consumption (as morphine equivalents) in the first 24 hours were recorded.

Quadriceps strength assessment

Quadriceps muscle strength was assessed using the Oxford Muscle Strength Scale (0-5) (Medical Research Council grading system) at six, 12, and 24 hours postoperatively [14]. Strength assessments were performed only after confirmed regression of SA, including recovery of lower limb sensation and motor function, to ensure measurements reflected block-related motor effects rather than residual neuraxial anesthesia. The Oxford score, therefore, represents functional quadriceps strength at the time of evaluation, not persistent SA. Assessments were performed using standardized instructions by blinded evaluators. Patients were assisted into a supported seated position at the bedside under continuous supervision, without weight-bearing or unsupported sitting. Knee extension was tested against graded manual resistance to ensure patient safety and adherence to institutional fall-prevention protocols. All patients completed strength assessments at each time point, and no missing or censored data were observed. The Oxford Muscle Strength Scale was chosen as a pragmatic bedside tool for early postoperative assessment of quadriceps function. Although volitional, assessments were conducted only after adequate analgesia and using standardized instructions to minimize pain-related inhibition. Evaluations were performed by trained anesthesiologists blinded to group allocation. A formal preoperative baseline motor assessment was not feasible due to fracture-related pain and immobilization; however, randomization ensured comparable baseline characteristics across groups.

Outcome measures

Baseline pain scores were recorded at rest and during passive limb movement prior to block administration. The primary outcome, reduction in VAS score at 30 minutes, was assessed 30 minutes after block placement and before SA. Postoperative VAS scores were measured at three, six, 12, and 24 hours, with time 0 defined as the time of block administration. The primary outcome was the difference in median VAS score reduction during movement at 30 minutes following the block. Secondary outcomes included the EOSP score; VAS at three, six, 12, and 24 hours postoperatively; time to first demand for rescue analgesia; 24-hour opioid consumption; patient satisfaction score; quadriceps muscle strength at six, 12, and 24 hours; and adverse events. Patient satisfaction prior to discharge was assessed categorically as “satisfied” or “dissatisfied,” based on overall pain reduction and comfort during positioning for SA, rather than using a numerical scale, owing to the advanced age and acute clinical condition of many participants.

Statistical analysis

Analysis was performed using IBM SPSS Statistics for Windows, Version 29.0 (Released 2022; IBM Corp., Armonk, NY, USA). Continuous variables were tested for normality using the Shapiro-Wilk test. Because most continuous outcomes were not normally distributed, results are reported as medians with IQRs and analyzed using the Kruskal-Wallis H test. The resulting p-values represent overall comparisons among the three groups. When the Kruskal-Wallis test was statistically significant, post hoc pairwise comparisons were conducted using the DSCF procedure to identify differences between specific pairs of groups. The DSCF method intrinsically controls the family-wise error rate and adjusts for multiple comparisons; no additional Bonferroni correction was applied. For key nonparametric outcomes, Hodges-Lehmann median differences with 95% CIs were calculated to estimate effect size. Effect size for Kruskal-Wallis tests was expressed as epsilon-squared (ε²), representing the proportion of variability in ranked outcomes attributable to group differences. An ε² value of approximately 0.01 indicates a small effect, 0.06 a moderate effect, and ≥0.14 a large effect. Categorical variables were expressed as counts and percentages. The chi-square test was used when all expected cell frequencies were ≥5; Fisher’s exact test was applied when one or more expected cell frequencies were <5. Accordingly, the chi-square test was used for variables such as sex distribution, ASA physical status, fracture type, and patient satisfaction, whereas Fisher’s exact test was used for sparse data, including adverse events and selected postoperative quadriceps muscle strength categories. Statistical significance was established at p < 0.05.

## Results

**Figure 5:**
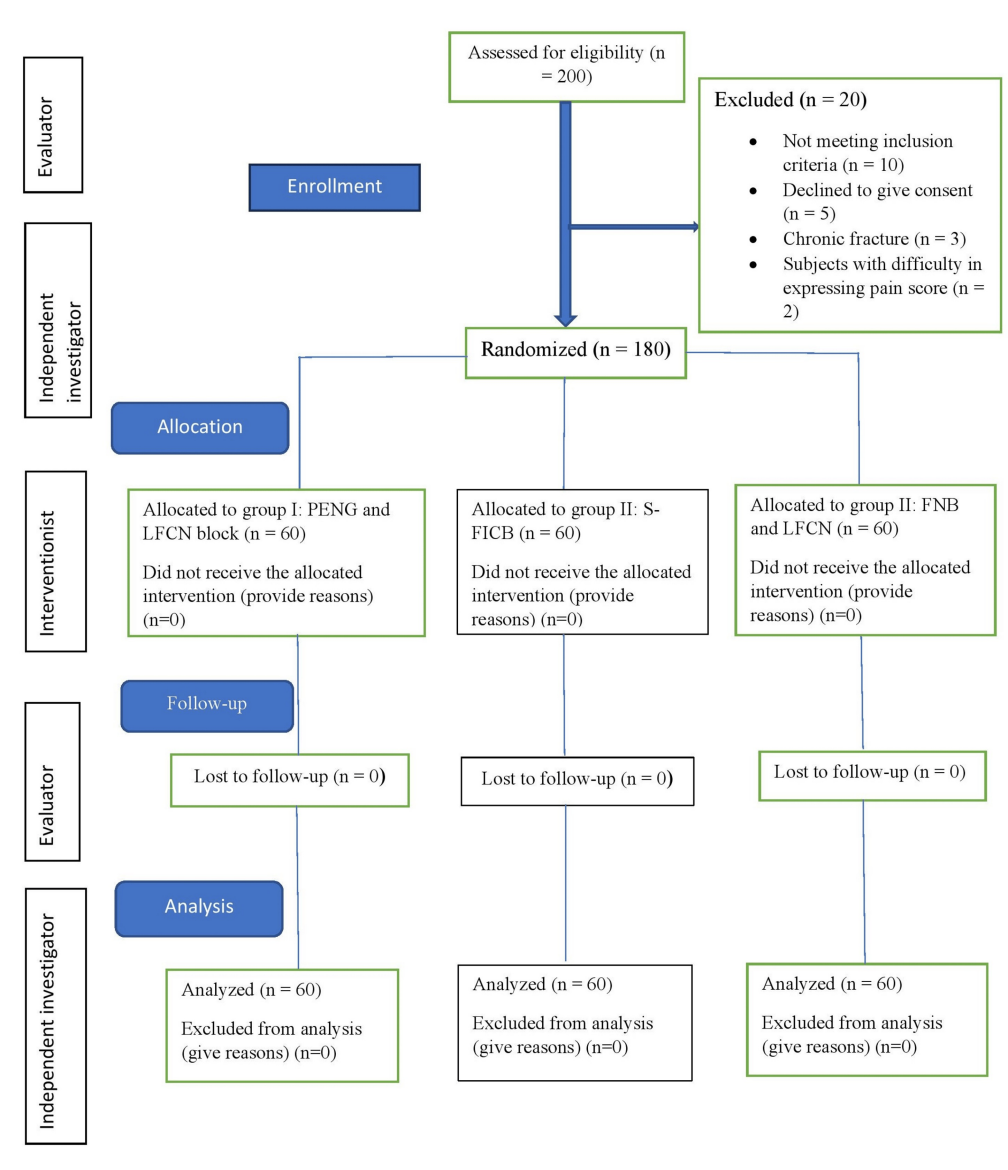
CONSORT flow diagram for enrollment, group allocation, follow-up, and analysis CONSORT, Consolidated Standards of Reporting Trials; FICB, fascia iliaca compartment block; FNB, femoral nerve block; LFCN, lateral femoral cutaneous nerve; PENG, pericapsular nerve group block

Of the 200 eligible subjects assessed, 20 patients were excluded due to not meeting the inclusion criteria (n = 10), refusal to provide consent (n = 5), chronic fractures (>7 days) (n = 3), or difficulty reporting pain scores (n = 2). The remaining 180 eligible patients were randomized into three groups (n = 60 each). All randomized patients received the allocated intervention, completed follow-up, and were included in the final analysis (Figure 5).

Baseline demographic and clinical characteristics were comparable across the three groups, including age, sex distribution, BMI, ASA physical status, fracture type (neck of femur vs. intertrochanteric), duration of surgery, and baseline VAS scores at rest and during movement. No statistically significant differences were observed among the groups for any baseline variable (Table 1).

**Table 1:** Baseline demographic and clinical characteristics Data are presented as median (IQR) or number (percentage), as appropriate. Continuous variables were compared using the Kruskal-Wallis test, with the H statistic reported. Categorical variables were analyzed using the Pearson chi-square test, with the χ² statistic reported. A p-value < 0.05 was considered statistically significant. ASA, American Society of Anesthesiologists; FICB, fascia iliaca compartment block; FNB, femoral nerve block; IT, intertrochanteric; LFCN, lateral femoral cutaneous nerve; NOF, neck of femur; PENG, pericapsular nerve group; VAS, Visual Analog Scale; VAS-M, Visual Analog Scale during movement; VAS-R, Visual Analog Scale at rest

Variable	PENG + LFCN (n = 60)	FICB (n = 60)	FNB + LFCN (n = 60)	Test statistic (H/χ²)	p-Value
Age (years)	68 (60-76)	69 (60-77)	66 (58-75)	H = 0.69	0.707
Female sex, n (%)	31 (51.7)	31 (51.7)	31 (51.7)	χ² = 0.00	1.000
BMI (kg/m²)	25.0 (21.5-26.2)	25.0 (22.2-26.2)	25.1 (22.2-26.3)	H = 0.07	0.967
ASA I, n (%)	15 (25.0)	15 (25.0)	10 (16.7)	χ² = 0.80	0.670
ASA II, n (%)	34 (56.7)	31 (51.7)	34 (56.7)	χ² = 0.80	0.670
ASA III, n (%)	11 (18.3)	14 (23.3)	16 (26.6)	χ² = 0.80	0.670
Duration of surgery (min)	90 (80-100)	90 (80-100)	90 (78-100)	H = 1.94	0.379
Fracture type - NOF, n (%)	32 (53.3)	33 (55.0)	31 (51.7)	χ² = 0.13	0.935
Fracture type - IT, n (%)	28 (46.7)	27 (45.0)	29 (48.3)	χ² = 0.13	0.935
Baseline VAS at rest (VAS-R)	6 (5-7)	6 (5-7)	6 (5-7)	H = 1.36	0.507
Baseline VAS on movement (VAS-M)	9 (8-10)	9 (9-10)	10 (9-10)	H = 4.07	0.129

**Figure 6:**
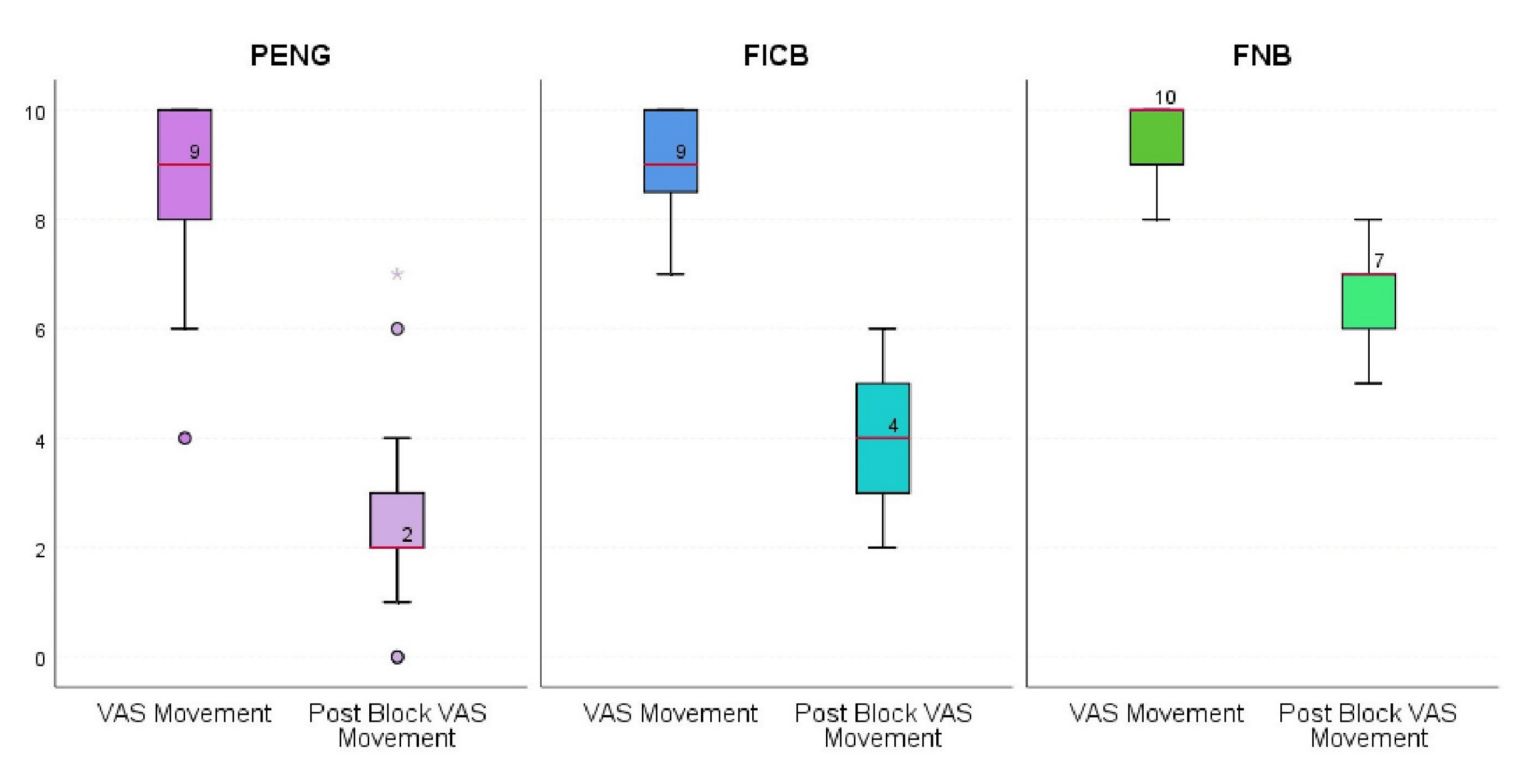
Box-and-whisker plot depicting baseline VAS during movement and post-block VAS at 30 minutes during movement among the three groups Each box plot contains the IQR, representing the middle 50% of the data (from Q1 to Q3). The line inside the box represents the median VAS score, and the “X” mark represents the mean VAS score. Whiskers indicate the range of scores excluding outliers, while dots represent outliers (values outside 1.5 times the IQR). Baseline VAS scores during movement were comparable across groups (median 9.5 (IQR 8-10), 9.5 (IQR 9-10), and 9.5 (IQR 9-10); p = NS). At 30 minutes post-block, median VAS scores decreased to 2 (2-3) in the PENG group, 4 (3-5) in the FICB group, and 7 (6-7) in the FNB group. The reduction in VAS was significantly different among the groups (Kruskal-Wallis H = 143.4, p < 0.0001). Post hoc pairwise comparisons showed a considerably greater analgesic effect with PENG than with FICB (p < 0.0001) and FNB (p < 0.0001). At the same time, FICB also achieved a greater reduction than FNB (p < 0.0001). FICB, fascia iliaca compartment block; FNB, femoral nerve block; LFCN, lateral femoral cutaneous nerve; PENG, pericapsular nerve group; VAS, Visual Analog Scale

At 30 minutes post-block, the median reduction in VAS scores at rest and during movement was significantly greater in the PENG + LFCN group than in the FICB and FNB + LFCN groups (p < 0.001) (Figure 6).

The Hodges-Lehmann median difference between PENG and FICB was 2.0 VAS points (95% CI: 1.5-2.5), and between PENG and FNB was 4.0 VAS points (95% CI: 3.5-4.5). The 30-minute post-block median VAS during movement was 2 (2-3) in the PENG + LFCN group, 4 (3-5) in the FICB group, and 7 (6-7) in the FNB + LFCN group. The 30-minute post-block median VAS at rest was 0 (0-1) in the PENG + LFCN group, 1 (1-2) in the FICB group, and 3 (2-4) in the FNB + LFCN group (Table 2).

**Table 2:** Primary analgesic outcomes at 30 minutes post-block Data are presented as median (IQR). Overall comparisons were performed using the Kruskal-Wallis test. Post hoc pairwise comparisons were conducted using the Dwass-Steel-Critchlow-Fligner test. The test statistic column reports the Kruskal-Wallis H statistic. Small letters indicate statistically significant pairwise differences: ᵃ, significantly different from the PENG + LFCN group; ᵇ, significantly different from the FICB group; ᶜ, significantly different from the FNB + LFCN group. For each significant pairwise comparison, the letter corresponding to the smaller category (ᵃ, ᵇ, or ᶜ) appears in the category with the larger median value. FICB, fascia iliaca compartment block; FNB, femoral nerve block; LFCN, lateral femoral cutaneous nerve; PENG, pericapsular nerve group block; VAS, Visual Analog Scale; VAS-M, Visual Analog Scale during movement; VAS-R, Visual Analog Scale at rest

Outcome	PENG + LFCN (n = 60)	FICB (n = 60)	FNB + LFCN (n = 60)	Test statistic (H)	p-Value
VAS-R reduction	0 (0-1)	1 (1-2)ᵃ	3 (2-4)ᵃᵇ	H = 143.40	<0.001
VAS-M reduction	2 (2-3)	4 (3-5)ᵃ	7 (6-7)ᵃᵇ	H = 143.40	<0.001
Change in VAS-M (ΔVAS-M)	7 (6-7)ᵇᶜ	5 (5-6)ᶜ	3 (3-3)	H = 143.40	<0.001

The PENG + LFCN block provided superior analgesia at rest and during movement at three, six, 12, and 24 hours postoperatively compared with FICB and FNB + LFCN (p < 0.001) (Table 3).

**Table 3:** Postoperative pain scores at rest and during movement Data are presented as median (IQR). p-Values represent overall group comparisons using the Kruskal-Wallis test. Post hoc pairwise comparisons were performed using the Dwass-Steel-Critchlow-Fligner test. The test statistic column reports the Kruskal-Wallis H statistic. Superscript letters indicate statistically significant pairwise differences: ᵃ, significantly different from the PENG + LFCN group and ᵇ, significantly different from the FICB group. For each significant pairwise comparison, the letter corresponding to the smaller category (ᵃ, ᵇ, or ᶜ) appears in the category with the larger median. FICB, fascia iliaca compartment block; FNB, femoral nerve block; LFCN, lateral femoral cutaneous nerve; PENG, pericapsular nerve group

Time point	PENG + LFCN	FICB	FNB + LFCN	Test statistic (H)	p-Value
Pain at rest					
Three hours	1 (0-1)	1 (1-2)ᵃ	2 (2-3)ᵃᵇ	81.74	<0.001
Six hours	1 (1-2)	2 (2-2)ᵃ	4 (3-4)ᵃᵇ	115.03	<0.001
12 hours	2 (1-2)	4 (3-4)ᵃ	4 (4-5)ᵃᵇ	113.02	<0.001
24 hours	2 (1-3)	3 (3-3)ᵃ	4 (3-4)ᵃᵇ	45.67	<0.001
Pain during movement					
Three hours	2 (1-2)	2 (2-3)ᵃ	4 (3-4)ᵃᵇ	112.28	<0.001
Six hours	2 (2-3)	3 (3-3)ᵃ	5 (5-6)ᵃᵇ	109.59	<0.001
12 hours	3 (3-4)	5 (5-6)ᵃ	7 (7-8)ᵃᵇ	122.82	<0.001
24 hours	4 (3-4)	4 (4-5)ᵃ	5 (4-6)ᵃᵇ	36.9	<0.001

The PENG + LFCN group had significantly higher EOSP scores than the FICB and FNB + LFCN groups (p < 0.001) (Table 4).

**Table 4:** EOSP Data are presented as counts. Overall comparisons were performed using the Kruskal-Wallis test or Fisher’s exact test, as appropriate. Post hoc pairwise comparisons were conducted using the Dwass-Steel-Critchlow-Fligner test. Fisher’s exact test was applied because of sparse cell counts; as this test does not generate a test statistic (χ²), only the exact p-value is reported. Small letters indicate statistically significant pairwise differences: a, significantly different from the PENG + LFCN group; b, significantly different from the FICB group; and c, significantly different from the FNB + LFCN group. For each significant pairwise comparison, the letter corresponding to the smaller category (ᵃ, ᵇ, or ᶜ) appears in the category with the larger median. EOSP, ease of spinal positioning; PENG, pericapsular nerve group

EOSP score	PENG + LFCN (n = 60)	FICB (n = 60)	FNB + LFCN (n = 60)	p-Value
1	0	0	55 (91.7)	<0.001
2	10 (16.7)	54 (90.0)ᵃᶜ	5 (8.3)	
3	50 (83.3)ᵇ	6 (10.0)	0	

The time to first analgesic demand was significantly prolonged in the PENG + LFCN group (p < 0.001), and 24-hour opioid consumption was significantly lower in this group compared with the other groups (p < 0.001) (Table 5). Patient satisfaction scores were also significantly higher in the PENG + LFCN group than in the FICB and FNB + LFCN groups (p < 0.001) (Table 5).

**Table 5:** Analgesic consumption and patient satisfaction Data are presented as counts. EOSP is an ordinal variable; however, Fisher’s exact test was used for overall comparisons because of sparse cell counts, and no rank-based post hoc testing was applied for this variable. The test statistic column reports the Kruskal-Wallis H statistic for continuous or ordinal variables and the Pearson chi-square (χ²) statistic for categorical variables. Small letters indicate statistically significant pairwise differences: ᵃ, significantly different from the PENG + LFCN group; ᵇ, significantly different from the FICB group; and ᶜ, significantly different from the FNB + LFCN group. For each significant pairwise comparison, the letter corresponding to the smaller category (ᵃ, ᵇ, or ᶜ) appears in the category with the larger median. EOSP, ease of spinal positioning; FICB, fascia iliaca compartment block; FNB, femoral nerve block; LFCN, lateral femoral cutaneous nerve; PENG, pericapsular nerve group

Variable	PENG + LFCN	FICB	FNB + LFCN	Test statistic	p-Value
24-hour opioid consumption (mg)	5 (0-5)	10 (10-10)ᵃ	20 (15-20)ᵃᵇ	H = 122.93	<0.001
Time to first rescue analgesia (hours)	24 (12-24)ᵇᶜ	12 (12-12)ᶜ	3 (3-6)	H = 107.12	<0.001
Patient satisfaction - Yes, n (%)	60 (100)	37 (61.7)ᶜ	20 (33.3)	χ² = 59.05	<0.001

The PENG + LFCN group demonstrated significantly less quadriceps weakness at six, 12, and 24 hours postoperatively than the FICB and FNB + LFCN groups (p < 0.001) (Table 6).

**Table 6:** Quadriceps muscle strength (Oxford Muscle Strength Scale) Data are presented as median (IQR). Overall comparisons were performed using the Kruskal-Wallis test or Fisher’s exact test, as appropriate. Post hoc pairwise comparisons were conducted using the Dwass-Steel-Critchlow-Fligner test. The test statistic column reports the Kruskal-Wallis H statistic. Small letters indicate statistically significant pairwise differences: ᵇ, significantly different from the FICB group and ᶜ, significantly different from the FNB + LFCN group. For each significant pairwise comparison, the letter corresponding to the smaller category (ᵃ, ᵇ, or ᶜ) appears in the category with the larger median. FICB, fascia iliaca compartment block; FNB, femoral nerve block; LFCN, lateral femoral cutaneous nerve; PENG, pericapsular nerve group

Time point	PENG + LFCN	FICB	FNB + LFCN	Test statistic (H)	p-Value
Six hours	4 (4-4)ᵇᶜ	0 (0-0)	0 (0-0)	H = 171.38	<0.001
12 hours	5 (5-5)ᵇᶜ	4 (3-4)ᶜ	3 (2-3)	H = 154.59	<0.001
24 hours	5 (5-5)ᵇᶜ	5 (4-5)	5 (4-5)	H = 42.70	<0.001

The p-values reported in the tables correspond to the overall Kruskal-Wallis test comparing the three groups. Statistically significant pairwise differences identified by post hoc Dwass-Steel-Critchlow-Fligner testing are indicated using lowercase letters (ᵃ, ᵇ, or ᶜ) to specify the groups involved.

## Discussion

Our trial compared the analgesic efficacy of three block techniques, such as PENG + LFCN, FICB, and FNB + LFCN, in patients with hip fractures. There was a substantial reduction in the 30-minute post-block VAS scores, both at rest and during movement, in the PENG + LFCN group compared with the FICB and FNB + LFCN groups. Our findings demonstrate that during movement, PENG produced the greatest reduction (7.0 points), followed by FICB (5.0 points) and FNB (3.0 points), with statistically significant intergroup differences. The results also demonstrated that the PENG + LFCN block provides superior analgesia, with significantly lower pain scores at rest and during movement at multiple time points in the 24-hour postoperative period compared with FICB and FNB + LFCN. The higher EOSP scores in the PENG + LFCN group indicate that this block facilitates easier patient positioning for SA, which is crucial for reducing patient discomfort and improving procedural efficiency. The longer time to first rescue analgesia and lower 24-hour opioid consumption in the PENG + LFCN group suggest that this block may help reduce opioid-related side effects and potentially enhance patient satisfaction. The minimal quadriceps weakness observed in the PENG + LFCN group is a significant advantage over the FICB and FNB + LFCN groups, as it may allow for earlier mobilization and potentially reduce the risk of falls. The higher satisfaction scores in the PENG + LFCN group likely reflect improved pain management and reduced side effects.

A detailed literature review revealed that the PENG block has been compared with FNB or FICB in head-to-head trials; however, no study has compared all three commonly performed blocks. In this respect, our study is unique. With suprainguinal FICB, the likelihood of LFCN blockade is high with 40 mL of local anesthetic; therefore, we included LFCN in both the FNB and PENG groups to ensure that all blocks were comparable [15,16]. Moreover, we evaluated both the analgesic profile and functional outcomes, unlike other trials that focused on either outcome alone.

The complexity of hip joint innervation, supplied by multiple nerves, poses a challenge for effective pain management. The sensory innervation of the anterior capsule of the hip comprises articular branches from the femoral nerve, obturator nerve (ON), and accessory ON (AON) [17]. The optimal RA technique for hip surgery should achieve a significant reduction in pain scores without delaying mobilization. Pain management following hip fracture has undergone a paradigm shift from FNB to FICB and, more recently, to the PENG block [4-6]. FICB and FNB primarily target the femoral nerve and may not achieve complete anesthetic coverage, as they do not consistently reach the ON. FNB is relatively straightforward to perform; however, it frequently spares regions innervated by the ON and LFCN. Furthermore, its advantages are counterbalanced by quadriceps weakness and an increased risk of falls [5,6]. FICB delivers the local anesthetic into a plane between the fascia iliaca and the underlying iliacus muscle. Evidence also indicates that FICB spares the ON to varying degrees [17]. With the advent of the PENG block, which targets the articular branches of the femoral nerve, ON, and AON supplying the anterior hip capsule, it represents an attractive alternative [7]. The LFCN block (in the PENG and FNB groups) targeted lateral thigh incisional pain [18].

Several recent systematic reviews have established that PENG provides superior analgesia in most included trials compared with FICB and FNB [19,20]. Pain relief at 30 minutes post-block was significantly greater in the PENG + LFCN group than in the FICB and FNB groups, consistent with previous studies [11,12,21]. VAS pain scores during the 24-hour postoperative period, both at rest and during movement, were reduced to a significantly greater extent in the PENG + LFCN group than in the FICB or FNB groups, in line with prior reports [22-26]. Likewise, the PENG + LFCN block resulted in significantly lower 24-hour opioid consumption and a prolonged time to first analgesic demand compared with FICB and FNB + LFCN [12,22,23,26-28].

Positioning for SA in the operating theater for patients with hip fractures is particularly challenging because of pain and muscle spasm in the hip region. In this regard, the ease with which patients were positioned for SA was significantly greater in the PENG + LFCN group than in the FICB and FNB groups. This EOSP benefit of the PENG block has been demonstrated in previous studies [3,11,21,27,29,30].

Superior preservation of quadriceps muscle strength in the PENG + LFCN group compared with the FICB and FNB + LFCN groups is an additional advantage. The greater preservation of quadriceps strength observed in the PENG group suggests a relatively short-term functional benefit during the early perioperative period. However, because motor assessment was performed at the bedside and follow-up was limited to 24 hours, these findings should be interpreted as early functional observations rather than definitive evidence of long-term recovery benefits. Preservation of strength may support safer positioning and early bedside activity, although the study was not designed to evaluate longer-term mobilization or rehabilitation outcomes. Early mobilization is strongly associated with a reduction in complications such as atelectasis, delirium, and particularly venous thromboembolism, which is a significant cause of morbidity in patients with hip fractures [4-6]. This distinguishes the PENG block from traditional FICB or FNB techniques [11,22,26,28,31]. No significant adverse effects were observed in any of the three groups.

Strengths and limitations

This study has several strengths that enhance the validity and clinical relevance of its findings. The randomized controlled design, adequate sample size, and standardized ultrasound-guided block techniques support strong internal methodological rigor. The inclusion of both analgesic and early functional outcomes provides a clinically meaningful assessment of perioperative performance. Blinded outcome assessment and prespecified endpoints further reduce measurement bias and strengthen interpretability. Comparison of three commonly used RA techniques adds practical relevance and contributes novel information to perioperative hip fracture analgesia.

Several limitations should be considered when interpreting these findings. First, the anesthesiologist performing the nerve blocks could not be blinded because of the nature of the interventions, introducing a potential risk of performance bias; however, postoperative assessments were conducted by blinded evaluators using standardized protocols. Second, quadriceps muscle strength was assessed using the Oxford scale, a pragmatic bedside tool that is inherently subjective and influenced by patient cooperation and therefore reflects functional motor performance rather than isolated neuromuscular blockade. Third, although rank-based post hoc testing controlled the family-wise error rate within comparisons, the assessment of multiple outcomes across several time points increases the possibility of type I error, and findings should be interpreted cautiously. Fourth, the study focused on early perioperative outcomes, and longer-term functional recovery, rehabilitation progress, and mobility were not evaluated. Finally, the single-center design and the absence of a formal preoperative motor baseline assessment may limit generalizability, although standardized protocols and balanced baseline characteristics support internal validity.

Clinical implications

The findings of this study suggest that a PENG-based regional analgesic approach may offer meaningful advantages during the early perioperative period in patients undergoing hip fracture surgery. Improved early analgesia and preservation of quadriceps strength may facilitate patient positioning for SA and enhance short-term comfort while reducing postoperative opioid requirements. These benefits are particularly relevant in frail or elderly patients, in whom minimizing opioid exposure and maintaining functional stability are important clinical goals. However, given the short follow-up period and the use of bedside functional assessment methods, these implications should be interpreted as applying primarily to early perioperative care rather than long-term rehabilitation outcomes. Further studies are warranted to determine whether these early advantages translate into sustained functional recovery.

## Conclusions

Ultrasound-guided PENG block combined with LFCN block provided superior early analgesia, facilitated easier positioning for SA, reduced postoperative opioid consumption, and better preserved quadriceps strength compared with fascia iliaca and FNBs in patients undergoing hip fracture surgery. These benefits were most evident during the early perioperative period, a clinically critical phase in this high-risk population. Although functional outcomes should be interpreted within the limitations of short follow-up and bedside motor assessment, the findings support a PENG-based approach as an effective motor-sparing regional analgesic technique. Larger multicenter studies with longer-term functional evaluation are warranted to confirm and extend these results.
